# Utero-placental (vascular) development throughout pregnancy in women with polycystic ovary syndrome: the Rotterdam Periconceptional Cohort

**DOI:** 10.1016/j.xfre.2024.12.002

**Published:** 2024-12-19

**Authors:** Lotte L. Berns, Rosalieke E. Wiegel, Anton H.J. Koning, Sten P. Willemsen, Joop S.E. Laven, Régine P.M. Steegers-Theunissen

**Affiliations:** a Department of Obstetrics and Gynecology, Erasmus MC University Medical Center, Rotterdam, the Netherlands; b Department of Pathology, Clinical Bioinformatics Unit, Erasmus MC University Medical Center, Rotterdam, the Netherlands; c Department of Biostatistics, Erasmus MC University Medical Center, Rotterdam, the Netherlands

**Keywords:** Polycystic ovary syndrome, pregnancy, placental development, first-trimester

## Abstract

**Objective:**

To study utero-placental vascular development from the first trimester onward in pregnant women with polycystic ovary syndrome (PCOS) and successful live births, compared with pregnant women without PCOS, after in vitro fertilization/intracytoplasmic sperm injection (IVF/ICSI) treatment or natural conception.

**Design:**

Prospective periconceptional cohort study in a single tertiary hospital.

**Subjects:**

Participants with ongoing pregnancies with available serial three-dimensional ultrasound scans were divided into 3 groups: women with PCOS; subfertile group, pregnancies who conceived via IVF/ICSI without PCOS; and fertile group, pregnancies who conceived naturally without PCOS.

**Exposure:**

PCOS diagnosis.

**Main Outcome Measures:**

During the first-trimester, placental volume (PV) and utero-placental vascular volume (uPVV) were measured offline in three-dimensional ultrasound volumes obtained at 7, 9, and 11 weeks’ gestational age (GA) using Virtual Organ Analysis and Virtual Reality. Serial measurements were obtained from uterine artery pulsatility and resistance indices (UtA PI and UtA RI) measured by pulsed-wave Doppler ultrasound as well as mean arterial pressure at 7, 9, 11, 13, 22, and 32 weeks’ GA. Similarly, the umbilical artery PI and RI were measured at 22 and 32 weeks’ GA.

**Results:**

We included 206 pregnancies in our study (PCOS n = 41; subfertile n = 63; fertile n = 102). Significantly negative associations were observed between PCOS and placental measurements (PV, uPVV, and their ratio) at 11 weeks’ GA with both the subfertile and fertile group as reference (e.g., uPVV 11 weeks’ GA: beta_PCOS-fertile_ –0.18 ∛cm^3^ [95% confidence interval: –0.30; –0.06]). UtA PI and RI were significantly lower throughout pregnancy in women with PCOS compared with the subfertile and fertile group. Women with PCOS showed a negative association with umbilical artery PI and RI at 32 weeks’ GA compared with the subfertile and fertile group as reference.

**Conclusion:**

Women with PCOS show decreased first-trimester placental development at 11 weeks’ GA compared with pregnancies without PCOS in the subfertile and fertile group. Additionally, these women also display lower UtA PI and UtA RI compared with women without PCOS. These results support the hypothesis that PCOS impacts early placental development, potentially contributing to adverse pregnancy outcomes. Further research should focus on the underlying pathophysiology and the modifying role of IVF/ICSI treatment.

Polycystic ovary syndrome (PCOS) is a prevalent endocrine disorder among reproductive-age women (1). Diagnosis is based on the 2003 Rotterdam Criteria, which require the presence of at least 2 of the 3 following characteristics: oligo/amenorrhea, hyperandrogenism, and polycystic ovaries (2). Following this definition, PCOS affects approximately 1 in every 10 women worldwide (3). The PCOS phenotype varies significantly due to diverse genetic and environmental factors. Frequently occurring characteristics in women with PCOS include obesity, insulin resistance, and elevated anti-müllerian hormone (AMH) levels (4, 5, 6), contributing to various comorbidities (7), such as diabetes mellitus type 2, cardiovascular disease, endometrial cancer, depression, and anxiety. Moreover, these women are at risk of both maternal pregnancy complications, including pregnancy-induced hypertension (PIH), preeclampsia (PE), and gestational diabetes mellitus, and neonatal complications, such as admission to a neonatal intensive care unit (8, 9, 10).

Placental dysfunction underlies many of these pregnancy complications, with the first trimester as an important window of placentation, starting 5–6 days after conception (11, 12, 13, 14, 15). Insufficient trophoblast invasion and inadequate uterine spiral arteries adaptation, alongside inflammation, characterize placental disorders, commonly observed in women with PCOS, indicating compromised utero-placental vascular development (16). These conditions align with the Barker hypothesis and DOHAD paradigm, suggesting suboptimal intrauterine conditions can affect long-term offspring health, potentially predisposing them to PCOS-like phenotypes due to prenatal androgen exposure (17).

Research has indicated elevated systolic and diastolic blood pressure throughout pregnancy and increased placental inflammation in women with PCOS (18, 19). Animal studies also demonstrate that adverse effects on fetal and placental weights, uterine artery blood flow, and vascular resistance are associated with PCOS (20).

From this background, we hypothesize that in pregnant women with PCOS, the utero-placental vascular development is compromised due to alterations in cardiovascular and metabolic pathways. Given the significance of the first trimester in placental development and the lack of studies focusing on PCOS and early placental development, this study aims to investigate utero-placental vascular development from the first trimester onward in pregnant women with PCOS with successful live births, compared with pregnant women without PCOS, after in vitro fertilization/intracytoplasmic sperm injection (IVF/ICSI) treatment or natural conception.

## Materials and methods

### Study design

Women in this study participated in the ongoing Rotterdam Periconceptional Cohort (Predict Study), conducted at the Department of Obstetrics and Gynaecology of the Erasmus Medical Centre Rotterdam (Erasmus MC) (21). This ongoing tertiary hospital–based prospective cohort includes pregnant women periconceptionally since 2009. A subcohort, with patients enrolled between January 2017 and March 2018, participated in the VIRTUAL Placenta study focusing on early placental development. Ethical approval was obtained from the Central Committee on Research in The Hague and the local Medical Ethical Committee of the Erasmus MC, and participants provided informed consent.

For this study we extracted data from the VIRTUAL Placenta study. Ongoing singleton pregnancies of less than 10^+0^ weeks of gestation were eligible for participation, provided that women were sufficiently fluent in the Dutch language. Exclusion criteria were miscarriage, oocyte donation, congenital malformations, and multiple pregnancies. The study involves pregnancies conceived naturally, after ovulation induction (OI) or mild ovarian hyperstimulation (MOH) with intrauterine insemination (IUI) or IUI alone, or after IVF treatment with or without ICSI. Standard protocols were used for assisted reproduction treatment including controlled ovarian stimulation, ovum pick-up, IVF treatment, and assessment of embryo morphology (22, 23). Indications for OI treatment included PCOS, whereas indications for MOH and/or IUI treatment were unexplained subfertility, male subfertility, or the need for sperm donation.

Participants visited the Erasmus MC at 7, 9, 11, 13, 22, and 32 weeks’ gestational age (GA), with questionnaires completed on enrollment. Diagnosis of PCOS according to the Rotterdam Criteria was accessed through initial screening, according to the 3 criteria described previously, at the outpatient clinic of the Erasmus MC by trained personnel (24). Ovulatory dysfunction was defined as amenorrhea (absence of menstrual bleeding >182 days) or oligomenorrhea (menstrual cycle >35 days or <8 cycles per year). Polycystic ovarian morphology was defined as ≥12 follicles in 1 or both ovaries (2–9 mm in diameter) and/or increased ovarian volume (>10 cm^3^) using an ultrasound at <8 MHz. Clinical hyperandrogenism was defined as a modified Ferriman-Gallwey score ≥5 (25). Biochemical hyperandrogenism was defined as a Free Androgen Index >4.5 and/or total testosterone >3.0 nmol/L. Participants who underwent the standardized screening and meeting the predefined criteria were subsequently diagnosed with PCOS.

### Study parameters

The questionnaires administered at enrollment collected demographic and personal data, including parity, preconceptional alcohol use, and medical history. The preconceptional period was defined as 14 weeks before conception up to 10 weeks after. Clinical visits involved 3D power Doppler ultrasound scans and pulsed-wave Doppler measurements of uterine and umbilical arteries at different GAs. Placental volume (PV) and utero-placental vascular volume (uPVV) were measured at 7, 9, and 11 weeks, whereas fetal circulation parameters (pulsatility and resistance index of the umbilical artery [UmbA PI and RI]) were monitored at 22 and 32 weeks. Maternal blood pressure and uterine artery measurements (PI and RI of the uterine artery [UtA PI and RI]) were measured at 7, 9, 11, 13, 22, and 32 weeks’ GA. Placenta-related complications, such as PIH, PE, preterm birth (PTB), fetal growth restriction (FGR), and small-for-gestational age (SGA) were diagnosed on the basis of clinical symptoms and confirmed by medical records. Hypertensive disorders were identified using the definition of the American College of Obstetricians and Gynecologists. FGR was determined on the basis of fetal abdominal circumference and/or estimated fetal weight below the 10th percentile, or a 20% decrease on the growth curve. PTB was defined as birth before 37 weeks, and SGA as birthweight below the 10th percentile adjusted for GA, parity, and fetal sex. Placental weight was measured and histologically examined postpartum by pathologists after standardized procedures according to the Amsterdam criteria.

### Ultrasound measurements

Measurements of PV and uPVV were collected using 3D power Doppler ultrasound scans of the vascular bed from the entire gestational sac including the placenta. The ultrasounds were performed by experienced researchers using a 6–12 MHz transvaginal probe of the GE Voluson E8 Expert system (GE, Zipf, Austria). Virtual Organ Computer-aided AnaLyis (VOCALTM) (4D View, GE Medical System, Zipf, Austria) was used to measure PV offline. The VOCAL algorithm was used to first create a 3D segmentation of the total uterus, including placenta and the gestational sac and subsequently a 3D segmentation of just the gestational sac. The PV could be calculated by subtracting the gestational sac volume from the total uterus volume. Power Doppler flow imaging was used to determine placenta vascularization by measuring the blood vessel volume using a virtual reality desktop system. On this system the V-Scope software was used to visually differentiate between the myometrium and placenta on the basis of echogenicity and power Doppler signal, allowing selection of the utero-placental vasculature after thresholding the PD signal. This has demonstrated to be a reliable manner of measuring the uPVV with intraclass correlation coefficients above 0.94 (26). We used standardized settings for the power Doppler ultrasound (power Doppler gain “–8.0,” pulse repetition frequency “0.6 kHz,” wall motion filter “low 1,” quality “high”). Moreover, we set the following default instrument settings: frequency, low; dynamic, set 5; balance, 180; smooth, 4/5; ensemble, 12; line density, 8; power Doppler map, 5; artifact suppression, on; power Doppler line filter, 1; quality, high. When bad image quality prevented the images from being examined properly, they were excluded from our measurements.

The UtA PI and RI were measured bilaterally at 7 till 13 weeks’ gestation using pulsed-wave Doppler ultrasound transvaginally and at 22 and 32’ weeks gestation transabdominally. The mean of the left and right uterine artery measurements was calculated and used for analysis.

### Study groups

Study participants were categorized by PCOS diagnosis and conception mode: women with PCOS (natural or OI/IUI or IVF/ICSI conception), subfertile women (IVF/ICSI conception), and fertile women without PCOS (natural or MOH/IUI conception). Additional analyses stratified the PCOS group by conception mode.

### Statistical analysis

Maternal and clinical baseline characteristics were summarized using mean ± standard deviation for normally distributed continuous variables, median and interquartile range for skewed continuous variables, and number with percentages for categorical variables, stratified by the 3 groups. Group differences were evaluated using unpaired two-sample Student’s *t*-tests, Wilcoxon rank sum tests, and Chi-squared tests, respectively. Pearson correlations were calculated between variables. Variable transformations were applied to establish normal distributions: cubic root for volumetric parameters (PV and uPVV) and natural log for nonvolumetric parameters (mean arterial pressure [MAP], UtA PI, UtA RI, UmbA PI, and UmbA RI). Parameters were analyzed using mean and standard deviation per measurement time point, with between-group differences assessed using Student’s *t*-tests. Linear models were estimated to examine the association between PCOS and utero-placental (vascular) development, adjusting the models for relevant confounders, was based on a literature search and differences at baseline. Model 1 was adjusted for GA. Model 2 was additionally adjusted for body mass index, nulliparity, and maternal age. Because of the relatively small sample size, it was not feasible to compare pregnancy complications between the PCOS and control groups within the analyses. However, given the high-risk profile of the fertile group within this tertiary care hospital cohort, we investigated the impact of placenta-related pregnancy complications by excluding the pregnancies complicated by PIH, PE, PTB, FGR, and SGA from analyses. Differences in placental weight were assessed using Student’s *t*-test. All analyses were performed in R Studio (R for Windows, version 3.6).

### PV measurements in the Predict Study

As a sensitivity analyses, we evaluated PV trajectories among the 3 study groups in the total cohort of the Predict Study (2010–2020). We evaluated associations between women with PCOS and PV with the same statistical methods and confounders as in the subcohort.

## Results

### Baseline characteristics

We enrolled 241 women, of whom 206 pregnancies were included in the analysis after applying exclusion criteria (Supplemental Fig. 1, available online). Reasons for exclusion included miscarriage (n = 22), congenital malformations (n = 8), oocyte donation (n = 4), and participant dropout (n = 1). The PCOS group consisted of 41 women, with 21 undergoing fertility treatment and 20 conceived naturally. The subfertile group included 63 couples undergoing IVF/ICSI, and the fertile group consisted of 102 couples conceiving naturally. Baseline characteristics show that women with PCOS were younger compared with the subfertile group and more often nulliparous than the fertile group (Table 1). Their preconceptional AMH levels were significantly higher than those of the subfertile group, without AMH data available for fertile women. Lifestyle factors such as smoking and alcohol consumption, resulting in phenotypes such as body mass index and baseline blood pressure, were not significantly different. There were no women with chronic pre-existing hypertension or use of antihypertensive medication. Newborn infants of women with PCOS were less frequently SGA compared with those in the subfertile group.

**Table 1:** Baseline characteristics of study participants stratified by fertility group.

Variable	PCOS (n = 41)	Subfertile (n = 63)	Fertile (n = 102)	*P*
Maternal age (y)	30.47 ± 3.66	34.05 ± 4.43	31.77 ± 4.42	<.001^a^^,^^b^
GA first visit (d)	58 (51–67)	54 (50–63)	57 (52–64)	.118
Nulliparous, n (%)	28 (68)	49 (78)	40 (39)	<.001^c^^,^^b^
Ethnicity, n (%)				
Dutch	34 (85)	51 (81)	74 (75)	.566
Western	0 (0)	2 (3.2)	4 (4.4)	.541
Non-western	6 (15)	10 (16)	21 (21)	.695
BMI at study entry (kg/m^2^)	25.32 (21.90–30.12)	24.24 (22.25–28.01)	25.21 (22.70–28.34)	.589
MAP at study entry (mm Hg)	81.41 ± 7.83	80.3 ± 7.66	79.94 ± 6.79	.553
Preconceptional alcohol use, n (%)	9 (22)	18 (29)	30 (29)	.657
Preconceptional smoking, n (%)	3 (7)	9 (14)	15 (15)	.473
Use of folic acid, n (%)	41 (100)	63 (100)	99 (97)	.214
Preconceptional AMH	8.2 (4.3–11.2)	4.2 (2.6–7.25)	NA	.033^a^
Educational level, n (%)				
Low	1 (2)	7 (11)	10 (10)	.697
Average	17 (43)	20 (32)	29 (29)	.536
High	22 (55)	36 (57)	60 (61)	.621
Mode of conception, n (%)				
IVF/ICSI	21 (51)	63 (100)	0 (0)	NA
Natural	20 (49)	0 (0)	102 (100)	NA
MOH/OI (PCOS)	4 (20.0)	NA	4 (3.9)	
IUI	2 (10.0)	NA	5 (4.9)	
Strictly dated pregnancies, n (%)	36 (88)	63 (100)	94 (92)	.468
Pregnancy complications, n (%)	5 (12)	14 (22)	34 (33)	
FGR	0 (0)	4 (6)	11 (11)	.076
PIH	2 (5)	2 (3)	5 (5)	.858
PE	1 (2)	3 (5)	3 (3)	.767
PTB	3 (7)	6 (10)	14 (14)	.486
SGA	1 (2)	3 (5)	15 (15)	<.024^c^

Data are presented as mean ± standard deviation (SD) for normal distributed variables, median, and interquartile range for skewed variables and number with percentages for categorical variables. Differences were analyzed using the unpaired two-sample *t*-test, two-sample Wilcoxon rank sum test and Chi-squared test, respectively. Preconceptional AMH is not applicable (NA) to the fertile group due to high amount of missing values.

AMH = anti-Müllerian hormone; BMI = body mass index; FGR = fetal growth restriction; GA = gestational age; ICSI = intracytoplasmic sperm injection; IUI = intrauterine insemination; IVF = in vitro fertilization; MAP = mean arterial pressure; MOH = mild ovarian hyperstimulation; OI = ovulation induction; PCOS = polycystic ovary syndrome; PE = preeclampsia; PIH = pregnancy-induced hypertension; PTB = preterm birth; SGA = small-for-gestational age.

a Statistical difference between PCOS and subfertile.

b Statistical difference between fertile and subfertile.

c Statistical difference between PCOS and fertile.

### First-trimester placental measurements

**Figure 1:**
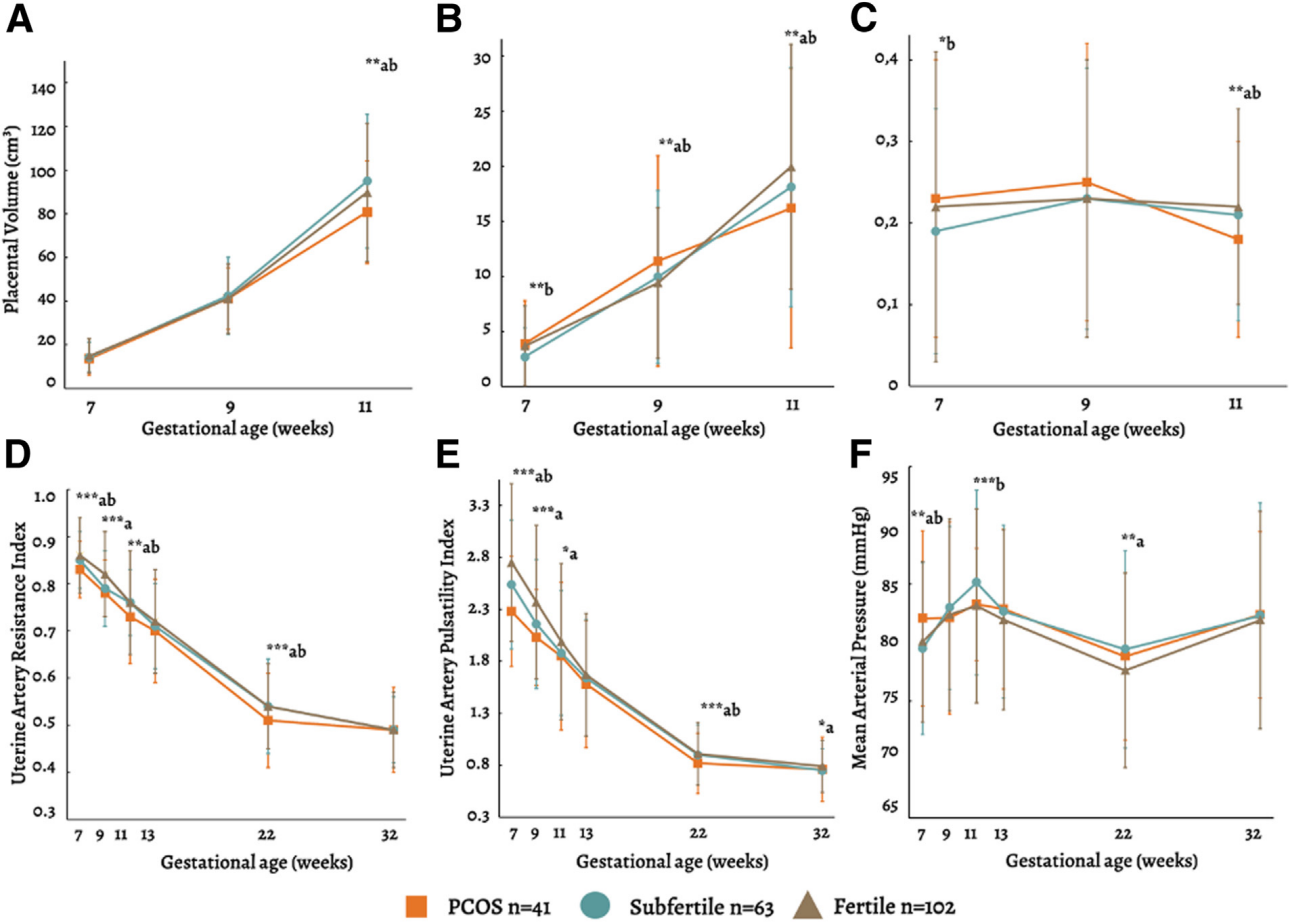
Line plots of (**A**) placental volume, (**B**) utero-placental vascular volume, (**C**) uPVV/PV ratio, (**D**) uterine artery resistance, (**E**) pulsatility indices, and (**F**) mean arterial pressure during pregnancy stratified by fertility groups. Mean ± SD. Differences were tested for significance using Student’s *t*-test at 7, 9, and 11 weeks’ GA for PV, uPVV, and uPVV/PVV ratio and 7, 9, 11, 13, 22, and 32 weeks’ GA for UtA PI and UtA RI. ∗∗∗*P*<.001, ∗∗*P*<.01, ∗*P*<.05. ^a^Statistical difference between PCOS and fertile group; ^b^Statistical difference between PCOS and subfertile group. Participants per group: PCOS N = 41; fertile N = 102; subfertile N = 63. GA = gestational age; PCOS = polycystic ovary syndrome; PV = placental volume; uPVV = utero-placental vascular volume; UtA PI = uterine artery pulsatility index; UtA RI = uterine artery resistance index.

During the first trimester, PV and uPVV increased in all groups. Figure 1 and Supplemental Table 1 (available online) present the crude differences in first-trimester placental development. The crude analysis and linear regression models yielded similar results; therefore, only the linear models will be discussed.

Linear regression models 1 and 2 indicated a significant negative association between PCOS and PV at 9 and 11 weeks’ gestation compared with the subfertile group (e.g., PV 11 weeks: beta_model 2_ PCOS-subfertile –0.286 ∛cm^3^; 95% confidence interval [CI]: –0.419; –0.152, *P*<.001) (Table 2). For uPVV, positive associations were observed with PCOS at 7 and 9 weeks’ GA, along with significantly negative associations at 11 weeks’ gestation compared with both the subfertile and fertile groups. Analyses for uPVV/PV ratio showed a positive association with PCOS at 7 weeks’ GA and negative associations at 11 weeks compared with the fertile group. After retransformation, PCOS exhibited an absolute difference of 12.4% (11.8 cm^3^) lower PV than the fertile group and a 17.3% (17.4 cm^3^) lower PV than the subfertile group at 11 weeks’ gestation. Exclusion of placenta-related complications revealed an additional negative association between PCOS and PV at 11 weeks’ GA compared with fertile women (*P*<.001). The analyses were repeated after stratifying the PCOS group into women who conceived naturally vs. via IVF/ICSI. The results remained the same when comparing women with PCOS conceived naturally with the fertile group, but vanished when comparing women with PCOS conceived through IVF/ICSI treatment with the subfertile group (Supplemental Table 2).

**Table 2:** Effect estimates for associations between utero-placental vascular development and fetal hemodynamic and PCOS throughout pregnancy by linear regression with the subfertile and fertile population as reference groups.

	PCOS – fertile	*P*	Model 2 – Β (95% CI)	*P*	PCOS – subfertile	*P*	Model 2 – Β (95% CI)	*P*
	Model 1 – Β (95% CI)				Model 1 – Β (95% CI)			
PV ($∛{\text{cm}}^{3}$)								
7 wk	–0.094 (–0.197; 0.008)	.071	–0.128 (–0.238; –0.017)	.024^a^	0.027 (–0.075; 0.128)	.604	0.097 (–0.017; 0.211)	.095
9 wk	0.031 (–0.039; 0.101)	.379	0.046 (–0.027; 0.118)	.215	–0.059 (–0.144; 0.026)	.173	–0.126 (–0.219; –0.032)	.009^a^
11 wk	–0.086 (–0.189; 0.017)	.101	–0.056 (–0.164; 0.053)	.313	–0.209 (–0.322; –0.095)	<.001^a^	–0.286 (–0.419; –0.152)	<.001^a^
uPVV ($∛{\text{cm}}^{3}$)								
7 wk	0.018 (–0.088; 0.124)	.736	0.069 (–0.042; 0.181)	.221	0.206 (0.097; 0.315)	<.001^a^	0.121 (0.007; 0.234)	.037^a^
9 wk	0.158 (0.065; 0.251)	<.001^a^	0.170 (0.079; 0.262)	<.001^a^	0.158 (0.057; 0.259)	.002^a^	0.148 (0.045; 0.252)	.005^a^
11 wk	–0.185 (–0.305; –0.063)	.003^a^	–0.181 (–0.301; –0.061)	.003^a^	–0.113 (–0.235; 0.009)	.070	–0.133 (–0.265; 0.000)	.049^a^
PVV/PV ratio								
7 wk	0.017 (–0.019; 0.054)	.346	0.045 (0.007; 0.084)	.021^a^	0.059 (0.021; 0.097)	.003^a^	0.017 (–0.023; 0.056)	.408
9 wk	0.027 (0.000; 0.054)	.053	0.025 (–0.002; 0.052)	.069	0.034 (0.005; 0.062)	.021^a^	0.014 (–0.017; 0.044)	.376
11 wk	–0.042 (–0.068; –0.016)	.002^a^	–0.032 (–0.059; –0.005)	.021^a^	–0.028 (–0.059; 0.003)	.079	–0.036 (–0.074; 0.002)	.067
Log UtA PI								
7 wk	–0.165 (–0.219; –0.110)	<.001^a^	–0.178 (–0.235; –0.120)	<.001^a^	–0.095 (–0.150; –0.039)	<.001^a^	–0.094 (–0.155; –0.032)	.003^a^
9 wk	–0.136 (–0.184; –0.089)	<.001^a^	–0.118 (–0.167; –0.070)	<.001^a^	–0.057 (–0.105; –0.009)	.021^a^	–0.050 (–0.103; 0.003)	.063
11 wk	–0.068 (–0.130; –0.006)	.033^a^	–0.062 (–0.126; 0.001)	.053	–0.046 (–0.102; 0.009)	.101	–0.014 (–0.074; 0.046)	.638
13 wk	–0.058 (–0.120; 0.004)	.068	–0.048 (–0.112; 0.017)	.146	–0.045 (–0.111; 0.020)	.177	–0.049 (–0.121; 0.022)	.179
22 wk	–0.120 (–0.169; –0.070)	<.001^a^	–0.097 (–0.148; –0.046)	<.001^a^	–0.088 (–0.147; –0.029)	.003^a^	–0.083 (–0.147; –0.020)	.010^a^
32 wk	–0.052 (–0.100; –0.004)	.034^a^	–0.040 (–0.089; 0.009)	.114	0.009 (–0.043; 0.061)	.737	0.003 (–0.054; 0.059)	.927
Log UtA RI								
7 wk	–0.042 (–0.059; –0.024)	<.001^a^	–0.040 (–0.059; –0.022)	<.001^a^	–0.022 (–0.038; –0.006)	.008^a^	–0.023 (–0.041; –0.005)	.013^a^
9 wk	–0.040 (–0.057; –0.024)	<.001^a^	–0.031 (–0.048; –0.014)	<.001^a^	–0.014 (–0.032; 0.004)	.123	–0.009 (–0.029; 0.010)	.350
11 wk	–0.035 (–0.060; –0.010)	.007^a^	–0.027 (–0.052; –0.001)	.039^a^	–0.037 (–0.057; –0.017)	<.001^a^	–0.025 (–0.047; –0.004)	.021^a^
13 wk	–0.021 (–0.049; 0.006)	.125	–0.013 (–0.041; 0.016)	.377	–0.020 (–0.047; 0.007)	.140	–0.022 (–0.051; 0.008)	.148
22 wk	–0.066 (–0.095; –0.038)	<.001^a^	–0.055 (–0.083; –0.026)	<.001^a^	–0.051 (–0.086; –0.015)	.005^a^	–0.048 (–0.086; –0.009)	.015^a^
32 wk	–0.011 (–0.040; 0.018)	.457	–0.002 (–0.031; 0.028)	.910	0.001 (–0.030; 0.031)	.972	–0.006 (–0.040; 0.027)	.706
Log UmbA PI								
22 wk	–0.002 (–0.025; 0.020)	.827	–0.005 (–0.028; 0.018)	.676	0.027 (0.003; 0.051)	.029^a^	0.034 (0.009; 0.059)	.008^a^
32 wk	–0.047 (–0.073; –0.021)	<.001^a^	–0.042 (–0.070; –0.015)	.002^a^	–0.056 (–0.086; –0.025)	<.001^a^	–0.076 (–0.109; –0.042)	<.001^a^
Log UmbA RI								
22 wk	–0.003 (–0.015; 0.010)	.670	–0.003 (–0.016; 0.010)	.640	0.010 (–0.004; 0.023)	.164	0.015 (0.001; 0.029)	.040^a^
32 wk	–0.031 (–0.048; –0.014)	<.001^a^	–0.029 (–0.046; –0.011)	.001^a^	–0.032 (–0.051; –0.012)	.001^a^	–0.045 (–0.066; –0.024)	<.001^a^
Placental weight (g)								
At birth	2.90 (–75.61; 81.41)	.941	8.20 (–70.03; 86.42)	.835	–104.78 (–214.62; 5.05)	.060	–161.67 (–289.15; –34.19)	.015^a^

Model 1: adjusted for GA. Model 2: adjusted for GA, maternal age, nulliparity, and BMI. PCOS N = 41; fertile N = 102; subfertile N = 63.

BMI = body mass index; CI = confidence interval; GA = gestational age; PCOS = polycystic ovary syndrome; PV = placental volume; UmbA PI = pulsatility index of the umbilical artery; UmbA RI = resistance index of the umbilical artery; uPVV = utero-placental vascular volume; UtA PI = pulsatility index of the uterine artery; UtA RI = resistance index of the uterine artery.

a *P*<.05.

### First-, second-, and third-trimester utero-placental vascular measurements

Women with PCOS showed consistently lower UtA PI and RI throughout pregnancy compared with both subfertile and fertile groups, particularly in the first and second trimester (Fig. 1; Supplemental Table 1). Multivariate linear modeling demonstrated significant negative associations between PCOS and UtA PI at 7, 9, and 22 weeks, gestation (e.g., [log] UtA PI 7 weeks: beta_model 2_ PCOS-fertile –0.178 [95% CI –0.235; –0.120], *P*<.001, Table 2). Similarly, significantly negative associations were found between PCOS and UtA RI at 7, 9, 11, and 22 weeks, gestation (e.g., [log] UtA RI 11 weeks GA: beta_model 2_ PCOS-fertile –0.027 [95% CI –0.052; –0.001], *P*=.039).

Significant positive associations between PCOS and MAP were observed during the second and third trimesters compared with the fertile group, after exclusion of placenta-related complications (e.g., [log] MAP (mmHg) 22 weeks GA: beta_model 2_ PCOS-fertile 0.020 [95% CI: 0.004; 0.035], *P*=.015) (Supplemental Table 3).

### Fetal circulation

**Figure 2:**
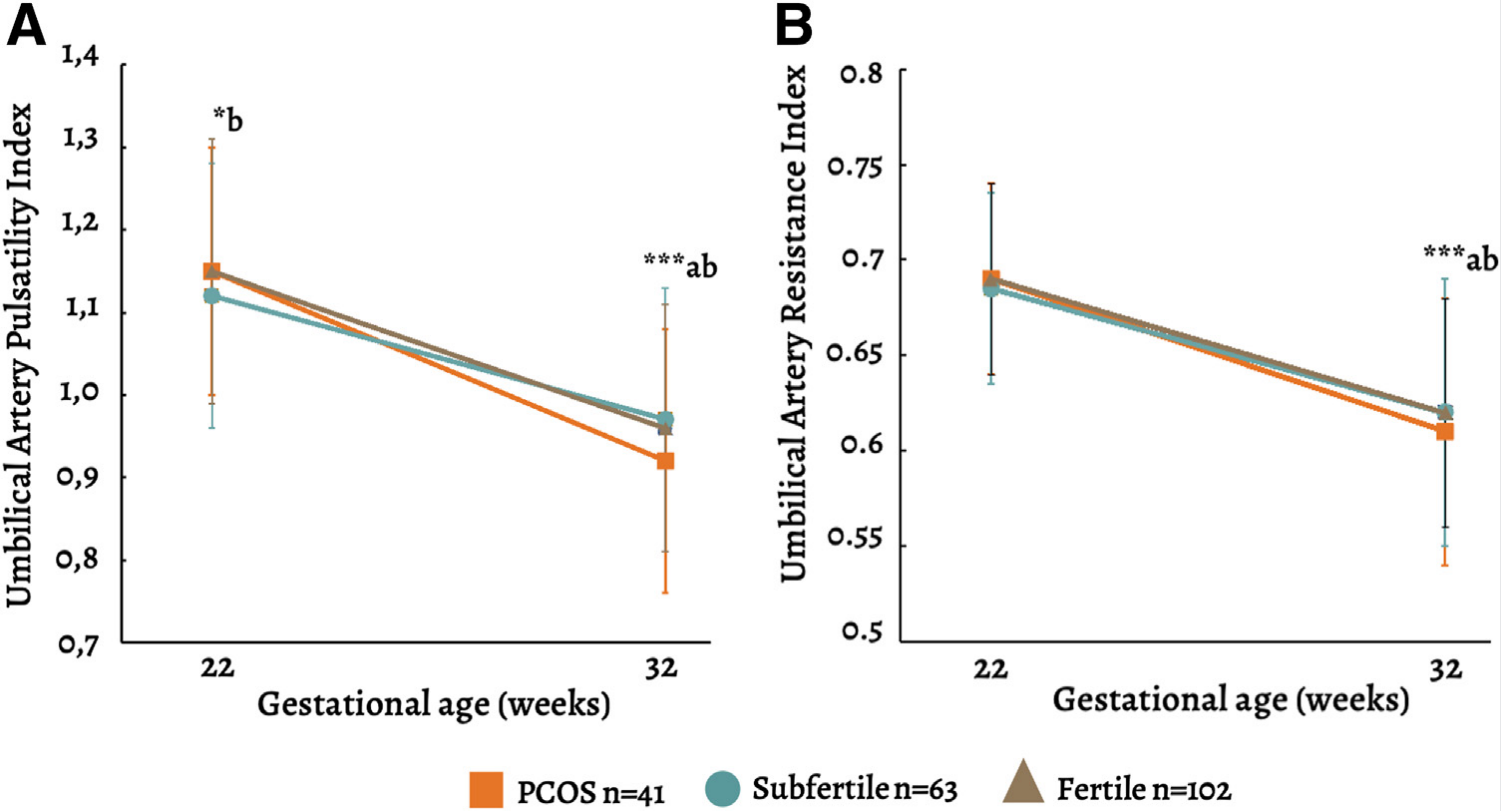
Line plots of umbilical artery (**A**) pulsatility indices and (**B**) resistance indices stratified by fertility group. Differences were tested for significance using Student’s *t*-test at 22 and 32 weeks’ GA. ∗∗∗*P*<.001, ∗*P*<.05. ^a^Statistical difference between PCOS and fertile; ^b^Statistical difference between PCOS and subfertile. Participants per group: PCOS N = 41; fertile N = 102; subfertile N = 63.GA = gestational age; PCOS = polycystic ovary syndrome; UmbA PI = pulsatility index of the umbilical artery; UmbA RI = resistance index of the umbilical artery.

The PCOS group showed significantly lower indices at 32 weeks compared with both fertile and subfertile groups (Fig. 2). Regression analyses confirmed this negative association. Moreover, PCOS exhibited a significant positive association with umbilical indices at 22 weeks, using the subfertile group as reference (e.g., [log] UmbA PI at 22 weeks GA: beta model 2 0.034 [95% CI: 0.009; 0.059], *P*=.008) (Table 2).

### Placental weight

Postpartum placental weight was available for 84 participants, including 14 with PCOS, 17 subfertile, and 53 fertile placentas. Mean placental weight was significantly lower in women with PCOS compared with the subfertile group (PCOS: 446.64 ± 139.11 g; subfertile: 563.53 ± 140.56 g, *P*=.009, Supplemental Table 1). This negative association was confirmed in multivariate linear modeling using the subfertile group as reference (Table 2). No significant differences were found compared with the fertile group.

### Sensitivity analysis: total cohort of Predict Study

First-trimester PV measurements were available for 882 women in the total Predict Study. Of these, 117 had PCOS, 336 were subfertile and conceived through IVF/ICSI treatment, and 429 were fertile and conceived naturally. The PCOS group exhibited significantly lower PV at 11 weeks’ GA compared with the subfertile group. Linear modeling confirmed this negative association using the subfertile group as reference. No significant differences were observed in PV between PCOS and the fertile group (Supplemental Table 4 and Supplemental Fig. 2). Given the high-risk nature of the fertile group within this cohort, we conducted an analysis excluding placenta-related complications. In this analysis, significant negative associations were observed between PCOS and PV at 11 weeks, with the fertile group as the reference (beta –0.234 $∛{\text{cm}}^{3}$ [95% CI: –0.452; –0.016], *P*=.036).

## Discussion

This study shows a negative association between first-trimester placental development (PV, uPVV, and uPVV/PV ratio) at 11 weeks’ gestation, and placental weight at birth in women with PCOS, compared with those without PCOS, both with and without known subfertility after IVF/ICSI treatment or natural conception. However, women with PCOS demonstrate lower uterine artery resistance indices throughout gestation and lower umbilical artery indices in the third trimester compared with the subfertile and fertile groups. Additionally, women with PCOS showed elevated blood pressure in early pregnancy. These findings support the hypothesis that PCOS may impact early placental development, potentially contributing to higher risks of placenta-related complications later in pregnancy.

To our knowledge, no previous studies have demonstrated impaired placental development in the first trimester of pregnancy in women with PCOS; however, postpartum placental weights have been shown to be lower in this population, which is consistent with our findings (27). The observed decreased placental vascular volume development at 11 weeks’ gestation and the absence of this at 7 weeks suggests a possible link with endometrium quality and vascularization in early pregnancy. A possible explanation for these alternating associations might be that angiogenesis might be stimulated in women with PCOS in early pregnancy as indicated by higher vascular endothelial growth factor and placental growth factor levels (28), whereas the capacity of the trophoblast to expand and their blood vessels to develop and dilate during the end of the first trimester might be impaired. Additionally, placentation up to approximately 9–10 weeks of gestation takes place in an environment without maternal-to fetal hemodynamic transfer due to trophoblastic plugs in the spiral arteries. We hypothesize that after dissolving these plugs the impaired utero-placental vascularization due to PCOS appears. Alternatively, dissolving of the plugs might take place earlier in women with PCOS, resulting in impaired placental development. Systemic cardiovascular, metabolic, or immunologic abnormalities during pregnancy adaptation in women with PCOS may contribute to the underlying pathology.

This study confirms prior research, indicating persistent elevated blood pressure throughout pregnancy in women with PCOS (18), potentially hindering the maternal cardiovascular adaptation to pregnancy. Interestingly, our findings reveal consistently lower uterine artery resistance indices in women with PCOS throughout gestation. Although this is in line with some of the limited number of previous studies, most of these show the opposite and support the common theories regarding the relation between placental development and uterine artery vascular adaptation (29, 30, 31). Although impaired placental development is typically associated with elevated uterine artery resistance (32), these study findings challenge this hypothesis, suggesting a more nuanced interplay between PCOS, cardiovascular adaptation, and placental development.

Vascular characteristics that are frequently observed in women with PCOS, such as overactivation of the renin-angiotensin aldosterone system (33, 34), are associated with disturbances in placental development and an increased risk of PE. Hypertension, commonly observed in PCOS (18), has correlations with placental dysfunction, inflammation, poor vascularization, and lower birth and placental weights (35). Additionally, chronic low-grade inflammation in PCOS, marked by elevated levels of C-reactive protein and interleukins (36, 37), can impact placental development and is linked to congenital heart anomalies and FGR (38, 39). Furthermore, altered metabolic profiles in PCOS, such as elevated AMH (5), homocysteine (40), and androgen levels (2), have previously been linked to FGR, impaired first-trimester placental development, PE, and decreased placental and fetal weights (6, 41, 42, 43, 44, 45). A recent study conducted by Dykgraaf et al. (28) revealed that elevated AMH levels during pregnancy were correlated with improved placental development and reduced vascular resistance. The latter observation is also seen in our PCOS group with high AMH levels. Additionally, because of anovulation, many women with PCOS display thickened endometrial linings due to an estrogen-predominant environment. The possible effect of estrogen levels is two-sided. An estrogen-predominant environment can initially support efficient placental vascularization and angiogenesis by promoting a well-developed endometrial lining. This could explain the improved placental vascular volume observed around 7 weeks in pregnant women with PCOS. However, excessive estrogen could potentially lead to complications in later stages of pregnancy, due to its effects on trophoblastic invasion and placental remodeling. An increase or decrease in placental size alone is not always a proxy for better or worse placental function, because endometrial size does not necessarily reflect the health and receptivity of the endometrium. Lastly, insulin resistance, frequently occurring in women with PCOS (4), is associated with higher birth weight, large for GA, and diverse adverse outcomes such as PTB (45, 46, 47). Impaired placental development might be a compensation mechanism to account for increased embryonic growth. Analysis of birth weight in this study revealed a trend that offspring of women with PCOS indeed had an increased birthweight (data not shown). In conclusion, multiple mechanisms, such as vascular, metabolic, and inflammatory factors, may collectively or independently contribute to impaired placental development in women with PCOS, and further research is needed to clarify these underlying relationships.

The importance of sufficient placental growth is accentuated by the Barker hypothesis and the later DOHAD paradigm, which suggest that suboptimal intrauterine circumstances can lead to the development of diseases later in life (17). Next to pregnancy and neonatal complications (8, 9, 10), later in life, children of mothers with PCOS are at increased risk for, among others, cardiovascular disease, reproductive and metabolic disorders, and autism spectrum disorders (10).

Strengths of this study are the prospective and longitudinal design within a single-center hospital-based setting, increasing internal validity and reducing heterogeneity. We validated our findings on PV in a larger cohort to increase the sensitivity and obtained the PCOS diagnosis from extensive standardized screening. However, participants were enrolled from a single-center tertiary hospital setting limiting generalizability to the population. Limitations include reduced generalizability due to the single-center design and potential underdiagnosis of PCOS, because some women may never have been tested for PCOS in the fertile study group.

However, participants were enrolled from a single-center tertiary hospital setting, which limits generalizability to the general population. Additional limitations include the potential underdiagnosis of PCOS, because some women in the fertile study group may never have been tested for this condition, and the adjusted GA estimated by ultrasound in pregnancies that were not strictly dated. Approximately 90% of PCOS and fertile pregnancies were strictly dated, whereas some pregnancies were estimated by ultrasound. However, additional modeling at 9 and 11 weeks’ GA, adjusting for the 7-week measurement, yielded consistent results. Caution is warranted in interpreting contradictory PV results at 7 weeks due to the lower sample size. Nonetheless, consistency was observed at 9 and 11 weeks in both models. Finally, residual confounding cannot be ruled out due to the observational nature of the study and the relatively small sample size of the subgroup analysis, which is therefore underpowered for sophisticated statistical analyses.

Although there were no women with pre-existing hypertension or use of antihypertensive medication, it remains important to consider factors such as insulin resistance, blood pressure, vascular resistance, and other cardiovascular parameters preconceptionally, because they can affect early placentation. Specifically, it would be useful to determine whether the metabolic or “classic” PCOS phenotype poses a greater risk. Additionally, other maternal exposures and conditions occurring between assessments could also have an impact.

Future research should focus on prospective pregnancy studies assessing placental development and biomarkers with preconception measurements of PCOS characteristics, particularly hyperandrogenism, impaired glucose tolerance, and additional metabolic and cardiovascular conditions, to clarify the underlying pathophysiologic pathways. Unhealthy lifestyle factors (e.g., smoking, alcohol use and malnutrition) also affect the maternal cardiovascular health and therefore early placentation. Continuous MAP and UtA assessments from preconception through the first trimester may be more sensitive than single baseline measurements. Our findings support investigating virtual reality placental parameters in a general population to assess first-trimester maternal hemodynamic adaptation. Comparing placental development in PCOS women who conceived naturally vs. those who underwent IVF/ICSI may reveal how assisted reproductive treatments impact pregnancy outcomes and fetal health (48, 49). In this study, significant differences were observed between naturally conceiving PCOS women and fertile women, but vanished between PCOS women who conceived with IVF/ICSI and subfertile women, possibly due to confounding factors and limited sample sizes. Heterogeneity in the IVF/ICSI group, including both fresh and cryopreserved embryo transfers, may also contribute to variability in outcomes. Extending these analyses to first-trimester 3D ultrasounds, including pregnancies ending in miscarriage or stillbirth, could improve insights into placental insufficiencies.

Understanding these pathways and the effects of conception mode on utero-placental development in PCOS patients may enhance pregnancy monitoring and reduce adverse outcomes. However, translating these findings into clinical practice requires further validation in a general population. Future research should focus on uncovering the underlying mechanisms linking PCOS with impaired utero-placental development, pregnancy course, and outcomes. Additionally, the focus should be toward the influence of maternal hemodynamic, cardiovascular, and endocrine health in patients with PCOS on placental development. This approach could eventually support the development of a risk assessment tool that integrates first-trimester placental measurements and maternal health factors, leading to early intervention and closer monitoring of pregnancies at risk.

## Conclusions

This study concludes that placental development is decreased at 11 weeks’ gestation in women with PCOS, possibly leading to lower placental weight at birth. Additionally, uterine and fetal artery resistance indices were reduced in women with PCOS. Previous studies highlighted that women with PCOS are at increased risk for pregnancy complications and adverse birth outcomes, and as such at risk for diseases later in life. These findings underline the importance to further investigate placental growth in women with PCOS. Ultimately, this might contribute to obtaining target points for early monitoring and pregnancy care optimization in women with PCOS.

## Declaration of Interests

L.L.B. has nothing to disclose. R.E.W. has nothing to disclose. A.H.J.K. has nothing to disclose. S.P.W. has nothing to disclose. J.S.E.L. has nothing to disclose. R.P.M.S.-T. has nothing to disclose.
