# Supplementary material for: Utero-placental (vascular) development throughout pregnancy in women with polycystic ovary syndrome: the Rotterdam Periconceptional Cohort
Source: F S Rep. 2024 Dec 19;6(1):79–89. doi: 10.1016/j.xfre.2024.12.002 (PMC11973822; doi:10.1016/j.xfre.2024.12.002)
Supplement: Supplemental Materials [file mmc1.docx]

**SUPPLEMENTAL MATERIALS**

**Utero-placental (vascular) development throughout pregnancy in women with polycystic ovary syndrome: the Rotterdam Periconceptional Cohort**

**Running title:** Placental development in PCOS patients

Lotte L. Berns^1*^, M.Sc; Rosalieke E. Wiegel^1*^, M.D. Ph.D; Anton H.J. Koning^2^, Ph.D; Sten P. Willemsen^1,3^, Ph.D; Joop S.E. Laven^1^, M.D. Ph.D; Régine P.M. Steegers-Theunissen^1^, M.D. Ph.D.

**Author affiliations:**

^1^Department of Obstetrics and Gynecology, Erasmus MC University Medical Center, Rotterdam, the Netherlands.

^2^Department of Pathology, Clinical Bioinformatics Unit, Erasmus MC University Medical Center, Rotterdam, the Netherlands.

^3^Department of Biostatistics, Erasmus MC University Medical Center, Rotterdam, the Netherlands.

*Both authors contributed equally

**Correspondence:**

Professor Régine P.M. Steegers-Theunissen, MD, PhD, Department of Obstetrics and Gynecology, Erasmus MC University Medical Center, Rotterdam, The Netherlands.

Postal address: Dr. Molewaterplein 40, 3015 GD Rotterdam, The Netherlands.

Phone: +31 10 7038254 Fax: +31 10 7036815

Mobile: +31 6 12472643 Email: r.steegers@erasmusmc.nl

**Article type**: Cohort study

**Funding:** This research was funded by the Department of Obstetrics and Gynaecology of the Erasmus MC University Medical Centre, Rotterdam, The Netherlands.


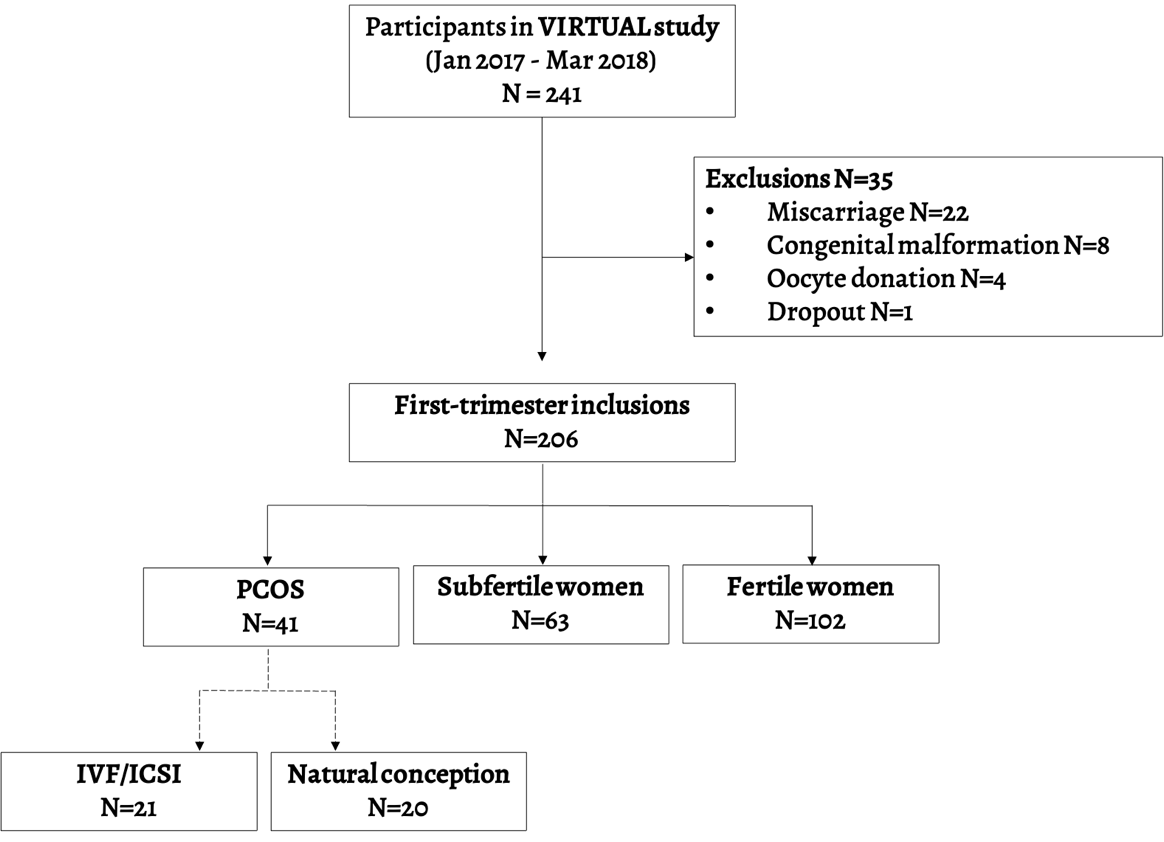


**Supplemental figure 1.** Flowchart of study participants. The PCOS group included OI (N=4) and IUI (N=2) and the fertile group included MOH+IUI (N=4) and IUI (N=5).

**Supplemental table 1.** Table of crude analysis of outcome variables stratified by fertility group

| Variable | PCOS (n=41) | Subfertile (n=63) | Fertile (n=102) | P-value |
| --- | --- | --- | --- | --- |
| PV ($\mathbf{cm}^{\mathbf{3}}$) |  |  |  |  |
| 7 wk | 16.45 ± 10.38 | 14.95 ± 6.64 | 17.80 ± 10.90 | 0.005^c^ |
| 9 wk | 45.64 ± 14.56 | 49.58 ± 22.63 | 44.60 ± 15.25 | 0.006^c^ |
| 11 wk | 89.78 ± 23.25 | 104.68 ± 38.94 | 97.15 ± 32.52 | 0.001^a,b,c^ |
| PVV ($\mathbf{cm}^{\mathbf{3}}$) |  |  |  |  |
| 7 wk | 3.90 ± 3.89 | 2.70 ± 2.62 | 3.70 ± 3.62 | <0.001^b,c^ |
| 9 wk | 11.39 ± 9.53 | 9.96 ± 7.83 | 9.41 ± 6.83 | 0.005^a,b^ |
| 11 wk | 16.19 ± 12.66 | 18.10 ± 10.84 | 19.93 ± 11.10 | 0.001^a,b^ |
| PVV/PV ratio |  |  |  |  |
| 7 wk | 0.23 ±0.17 | 0.19 ± 0.15 | 0.22 ± 0.19 | 0.019^b^ |
| 9 wk | 0.25 ± 0.17 | 0.23 ± 0.16 | 0.23 ± 0.17 | 0.181 |
| 11 wk | 0.18 ± 0.12 | 0.21 ± 0.13 | 0.22 ± 0.12 | 0.004^a,b^ |
| UtA PI |  |  |  |  |
| 7 wk | 2.28 ± 0.53 | 2.54 ± 0.62 | 2.75 ± 0.76 | <0.001^a,b,c^ |
| 9 wk | 2.03 ± 0.46 | 2.16 ± 0.62 | 2.37 ± 0.74 | <0.001^a,c^ |
| 11 wk | 1.85 ±0.71 | 1.88 ± 0.60 | 1.99 ± 0.75 | 0.029^a^ |
| 13 wk | 1.58 ± 0.61 | 1.64 ± 0.56 | 1.67 ± 0.59 | 0.104 |
| 22 wk | 0.82 ± 0.29 | 0.90 ± 0.29 | 0.91 ± 0.30 | <0.001^a,b^ |
| 32 wk | 0.76 ± 0.31 | 0.75 ± 0.21 | 0.79 ± 0.25 | 0.004^a,c^ |
| UtA RI |  |  |  |  |
| 7 wk | 0.83 ± 0.06 | 0.85 ± 0.06 | 0.86 ± 0.08 | <0.001^a,b,c^ |
| 9 wk | 0.78 ± 0.07 | 0.79 ± 0.08 | 0.82 ± 0.09 | <0.001^a,c^ |
| 11 wk | 0.73 ± 0.10 | 0.76 ± 0.07 | 0.76 ± 0.11 | 0.004^a,b^ |
| 13 wk | 0.70 ± 0.11 | 0.71 ± 0.09 | 0.72 ± 0.11 | 0.155 |
| 22 wk | 0.51 ± 0.10 | 0.54 ± 0.10 | 0.54 ± 0.09 | <0.001^a,b^ |
| 32 wk | 0.49 ± 0.09 | 0.49 ± 0.07 | 0.49 ± 0.08 | 0.516 |
| MAP (mmHg) |  |  |  |  |
| 7 wk | 82.05 ± 7.47 | 79.47 ± 7.33 | 80.07 ± 6.85 | 0.004^a,b^ |
| 9 wk | 82.09 ± 8.20 | 82.96 ± 6.96 | 82.36 ± 8.20 | 0.239 |
| 11 wk | 83.25 ± 4.78 | 85.11 ± 7.89 | 83.13 ± 8.28 | <0.001^b,c^ |
| 13 wk | 82.85 ± 6.83 | 82.64 ± 7.39 | 81.93 ± 7.67 | 0.153 |
| 22 wk | 78.81 ± 7.13 | 79.42 ± 8.42 | 77.61 ± 8.30 | 0.002^a,c^ |
| 32 wk | 82.36 ± 7.10 | 82.28 ± 9.63 | 81.91 ± 9.29 | 0.614 |
| UmbA PI |  |  |  |  |
| 22 wk | 1.15 ± 0.15 | 1.12 ± 0.16 | 1.15 ± 0.16 | 0.025^b,c^ |
| 32 wk | 0.92 ± 0.16 | 0.97 ± 0.16 | 0.96 ± 0.15 | <0.001^a,b^ |
| UmbA RI |  |  |  |  |
| 22 wk | 0.69 ± 0.05 | 0.68 ± 0.05 | 0.69 ± 0.05 | 0.158 |
| 32 wk | 0.61 ± 0.07 | 0.62 ± 0.07 | 0.62 ± 0.06 | <0.001^a,b^ |
| Placental weight (gram) |  |  |  |  |
| At birth | 446.6 ± 139.1 | 563.5 ± 140.6 | 452.2 ± 125.1 | 0.009^,b^ |

Differences were tested for significance using the Student’s t-test. ^a^ Statistical difference between PCOS and fertile; ^b^ Statistical difference between PCOS and subfertile; ^c^ Statistical difference between subfertile and fertile. Participants per group: PCOS N=41; fertile N=102; subfertile N=63. Abbreviations: MAP: Mean Arterial Pressure; PV: Placental Volume; uPVV: Utero-placental Vascular Volume; UtA PI: Pulsatility Index of the Uterine Artery; UtA RI: Resistance Index of the Uterine Artery; UmbA PI: Pulsatility Index of the Umbilical Artery; UmbA RI: Resistance Index of the Umbilical Artery.

**Supplemental table 2:** Effect estimates for associations between first-trimester placental volume, utero-placental vascular measurements and fetal circulation, and PCOS by using multivariate linear regression comparing PCOS+natural conception and PCOS+IVF with respectively the fertile and subfertile population as reference groups.

| **Variable** | **PCOS+ivf – subfertile** |  | **PCOS+natural – fertile** |  |
| --- | --- | --- | --- | --- |
|  | **Β (95% CI)** | **P-value** | **Β (95% CI)** | **P-value** |
| **PV (**$\boldsymbol{∛}\boldsymbol{cm}^{\boldsymbol{3}}$**)** |  |  |  |  |
| *7 wk* | -0.053 (-0.173; 0.067) | 0.383 | 0.201 (0.036; 0.365) | 0.017* |
| *9 wk* | 0.009 (-0.096; 0.113) | 0.873 | -0.002 (-0.107; 0.103) | 0.967 |
| *11 wk* | -0.075 (-0.231; 0.081) | 0.343 | -0.207 (-0.352; -0.062) | 0.005* |
| **PVV (**$\boldsymbol{∛}\boldsymbol{cm}^{\boldsymbol{3}}$**)** |  |  |  |  |
| *7 wk* | 0.067 (-0.058; 0.193) | 0.291 | 0.225 (0.071; 0.379) | 0.004* |
| *9 wk* | 0.231 (0.111; 0.350) | <0.001* | 0.066 (-0.060; 0.193) | 0.304 |
| *11 wk* | 0.042 (-0.120; 0.204) | 0.611 | -0.220 (-0.365; -0.076) | 0.003* |
| **PVV/PV ratio** |  |  |  |  |
| *7 wk* | 0.026 (-0.018; 0.070) | 0.246 | 0.049 (-0.010; 0.107) | 0.104 |
| *9 wk* | 0.036 (0.000; 0.073) | 0.052 | 0.005 (-0.031; 0.042) | 0.775 |
| *11 wk* | 0.006 (-0.034; 0.046) | 0.769 | -0.037 (-0.074; -0.001) | 0.044* |

All analyses performed with model 2: adjusted for GA, maternal age, nulliparity and BMI. * p<0.05. PCOS+IVF N=21; PCOS+natural = 20; fertile N=102; subfertile N=63. Abbreviations: GA: gestational age; PV: placental volume; uPVV: utero-placental vascular volume; CI: Confidence Interval.

**Supplemental table 3.** Effect estimates for associations between log transformed mean arterial pressure and PCOS throughout pregnancy by multivariate linear regression with the subfertile and fertile population as reference groups

|  | **PCOS – fertile** |  |  |  | **PCOS – subfertile** | | |  |
| --- | --- | --- | --- | --- | --- | --- | --- | --- |
|  | **Model 1** |  | **Model 2** |  | **Model 1** |  | **Model 2** |  |
|  | **Β (95% CI)** | **P-value** | **Β (95% CI)** | **P-value** | **Β (95% CI)** | **P-value** | **Β (95% CI)** | **P-value** |
| **Log MAP (mmHg)** |  |  |  |  |  |  |  |  |
| 7 wk | 0.018  (0.001; 0.036) | 0.044* | 0.014  (-0.003; 0.032) | 0.101 | 0.027  (0.006; 0.048) | 0.011* | 0.032  (0.010; 0.053) | 0.004* |
| 9 wk | -0.007  (-0.023; 0.009) | 0.364 | -0.004  (-0.020; 0.012) | 0.589 | -0.013  (-0.029; 0.003) | 0.104 | -0.010  (-0.026; 0.006) | 0.246 |
| 11 wk | 0.002  (-0.013; 0.017) | 0.763 | 0.001  (-0.014; 0.016) | 0.878 | -0.023  (-0.037; -0.009) | 0.001* | -0.024  (-0.038; -0.009) | 0.001* |
| 13 wk | 0.010  (-0.005; 0.025) | 0.189 | 0.019  (0.004; 0.034) | 0.012* | 0.000  (-0.015; 0.016) | 0.964 | 0.009  (-0.008; 0.026) | 0.299 |
| 22 wk | 0.012  (-0.005; 0.028) | 0.162 | 0.012  (-0.002; 0.027) | 0.095 | -0.011  (-0.029; 0.007) | 0.235 | -0.001  (-0.017; 0.014) | 0.881 |
| 32 wk | 0.002  (-0.015; 0.019) | 0.806 | 0.003  (-0.014; 0.019) | 0.744 | 0.002  (-0.017; 0.020) | 0.860 | 0.017  (-0.002; 0.036) | 0.077 |

Model 1: adjusted for GA. Model 2: adjusted for GA, maternal age, nulliparity and BMI. * p<0.05. PCOS N=41; fertile N=102; subfertile N=63. Abbreviations: MAP: mean arterial pressure.

**Supplemental table 4:** Effect estimates for associations between first-trimester placental volume and PCOS in the total cohort of the Predict study (2010-2020) by using multivariate linear regression with the subfertile and fertile population as reference groups.

|  | **PCOS – fertile** |  |  |  | **PCOS – subfertile** | | |  |
| --- | --- | --- | --- | --- | --- | --- | --- | --- |
|  | **Model 1** |  | **Model 2** |  | **Model 1** |  | **Model 2** |  |
|  | **Β (95% CI)** | **P-value** | **Β (95% CI)** | **P-value** | **Β (95% CI)** | **P-value** | **Β (95% CI)** | **P-value** |
| **PV (**$∛{cm}^{3}$**)** |  |  |  |  |  |  |  |  |
| 7 wk | -0.109  (-0.225; 0.006) | 0.063 | -0.110  (-0.228; 0.007) | 0.065 | -0.065  (-0.162; 0.031) | 0.183 | -0.074  (-0.178; 0.031) | 0.168 |
| 9 wk | 0.006  (-0.109; 0.121) | 0.915 | 0.012  (-0.105; 0.129) | 0.838 | -0.033  (-0.149; 0.082) | 0.570 | -0.087  (-0.211; 0.038) | 0.171 |
| 11 wk | -0.108  (-0.255; 0.040) | 0.151 | -0.101  (-0.259; 0.058) | 0.211 | -0.206  (-0.356; -0.055) | 0.008* | -0.247  (-0.408; -0.085) | 0.003* |

Model 1: adjusted for GA. Model 2: adjusted for GA, maternal age, nulliparity and BMI. * p<0.05. PCOS N=117; fertile N=429; subfertile N=336. Abbreviations: GA: gestational age; PV: placental volume; CI: Confidence Interval


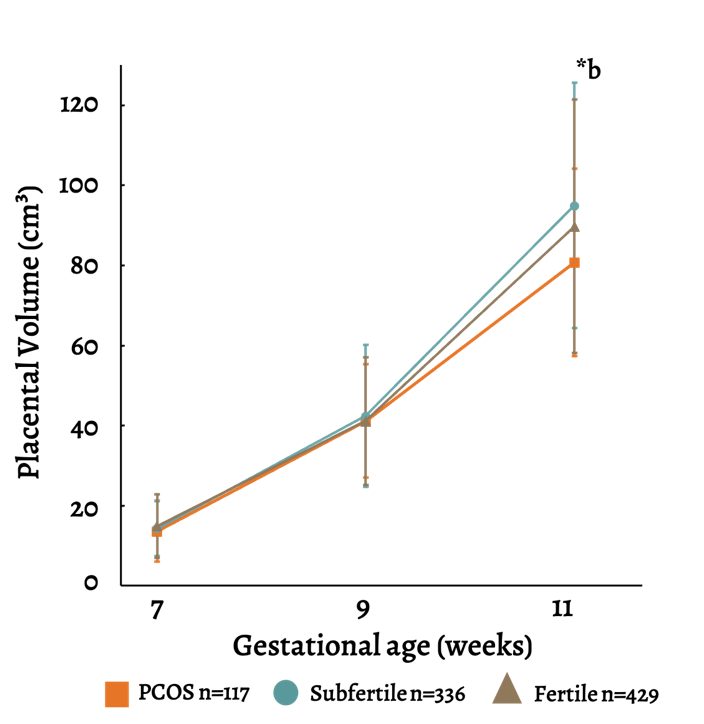


**Supplemental figure 2:** Line plot of placental volume in the total cohort of the Predict study stratified by fertility groups. Differences were tested for significance using the Student’s t-test at 7, 9 and 11 weeks GA. *p<0.05. ^b^ Statistical difference between PCOS and subfertile. Participants per group: PCOS N=117; fertile N=429; subfertile N=336.
